# Using Free and Open-Source Bioconductor Packages to Analyze Array Comparative Genomics Hybridization (aCGH) Data

**DOI:** 10.2174/138920209787581244

**Published:** 2009-03

**Authors:** Simon Lin, Pan Du, Nadereh Jafari, Toru Ouchi

**Affiliations:** Feinberg School of Medicine, Northwestern University, Chicago, IL 60611, USA

## Abstract

Whole-genome array Comparative Genomics Hybridization (aCGH) can be used to scan chromosomes for deletions and amplifications. Because of the increased accessibility of many commercial platforms, a lot of cancer researchers have used aCGH to study tumorigenesis or to predict clinical outcomes. Each data set is typically in several hundred thousands to one million rows of hybridization measurements. Thus, statistical analysis is a key to unlock the knowledge obtained from an aCGH study. We review several free and open-source packages in Bioconductor and provide example codes to run the analysis. The analysis of aCGH data provides insights of genomic abnormalities of cancers.

## INTRODUCTION

DNA copy number alternations are critical events in the development and progression of cancers [1]. The array Comparative Genomics Hybridization (aCGH) provides a rapid screening of the whole genome for deletions and amplifications along chromosomes.

For instance, in our current studies, we use Sentrix HumanHap650Y Genotyping BeadChips from Illumina. Tag SNPs on these chips interrogates >660,000 SNPs that can be used for whole-genome association studies and LOH/Copy number analyses. The Tag SNPs on the chip were selected from the HapMap release 21 data set. On average, the 650Y chips have one common SNP (MAF>0.05) every 5.3kb, 6.2kb, and 5.4kb across the genome in the CEU, CHB+JPT, and YRI populations, respectively. The average 90^th^ percentile gap between common SNPs (MAF>0.05) on the Human Hap650Y BeadChip is 12kb, 14kb and 12kb in the CEU, CHB+JPT, and YRI populations, respectively. As such, if we conservatively estimate that 10 SNP markers are necessary to detect an amplified or deleted region, we can use the Hap650Y BeadChip to detect chromosomal alterations at a resolution of 120-140 kb.

Briefly, aCGH has been widely used in cancer studies to detect genetic alterations, although aCGH usually cannot detect translocations and inversions. aCGH has been used for the following two goals:

### 1) To Study Tumorigenesis

Genetic alterations, wherever measured by aCGH, can be used to find potential oncogenes (in amplified regions) and tumor suppressor genes (in deleted regions). For instance, five candidate tumor suppressor genes were discovered in a glioma study [2]. Moreover, the genetics alterations can be traced over time to monitor the progression of tumors.

### 2) To Predict Clinical Outcomes

Because tumorigenesis is driven by sequential acquisition of genetic alterations, it is highly likely that genomic aberration patterns can be used to predict tumor subtype, grade, or patient survival. Indeed, numerous studies have shown the potential of using aCGH in predicting clinical outcomes. For instance, a 190-gene signature was shown to predict astrocytic glioma with 92% accuracy [2]. However, these studies were usually retrospective rather than prospective. In addition, only one site was usually involved in the processing of the microarrays. Although the cross-institute and cross-platform consistency has been shown with RNA profiling methods using microarrays [3], it is still unclear how robust the findings from aCGH are.

#### aCGH

Earlier aCGH studies usually used home-made arrays with BAC clones. It was tedious, low resolution (because of the BAC clones), and hard to control the array-printing quality. Recent works extensively use commercial designs of oligo arrays from Agilent, Affymetrix and Illumina. It is interesting to note that Affymetrix and Illumina do not have any product lines of aCGH; aCGH was only considered as an “off-label” use of the SNP arrays. As such, the Affymetrix and Illumina platforms are able to genotype and to detect the chromosome aberrations at the same time. Because it is relatively common for these SNP microarrays to have up to one million markers for the whole human genome, aCGH can be achieved at a very high resolution. A higher resolution can help one to see the boundaries of chromosomal alteration more precisely. A more elaborate introduction of aCGH can be found in a recent review paper [4].

After data preprocessing, regardless of the chosen platform, the aCGH data can be reported as (x,y) pairs: x is the chromosome location; y is the normalized value proportional to the DNA copy number. Normally human cells contain two copies of each of the 22 non-sex chromosomes. Any deviations in the DNA copy number will result in altered y values. The objective of the data analysis is to identify the regions of gains and losses along the chromosome.

## EVALUATION OF aCGH SEGMENTATION ALGORITHMS

Here we use a simulated data to illustrate how to run aCGH analysis using free and open-source packages in Bioconductor (Table **1**). Bioconductor is a collection of statistical analysis packages for genomics data [5]. Here we selected four popular aCGH analysis packages: aCGH [6], snapCGH [7], DNAcopy [8] and GLAD [9]. A brief description of the packages is shown in Table **1**.

Fig. (**1**) shows the computer codes to run each segmentation algorithm and one representative result. The simulated data (500 data points) has both varying segment lengths and varying amplitudes, as shown in Fig. (**1**). As the snapCGH package provides interface functions for the segmentation algorithms of different packages, we used these functions to run the simulation data. Note that the efficient design of the snapCGH package makes the programming as easy as one line of codes.

A visual examination of Fig. (**1**) suggests that the performance of different algorithms is similar. To better evaluate the difference in performance, we ran 100 simulated aCGH data for both varying segment amplitudes and fixed segment amplitudes, and then evaluate the difference between the estimated segmentation and true segmentation. Table **2** shows the summary of the evaluation results. We can see the HMM-based algorithms have better performances for the simulation data with fixed amplitudes. The major reasons include HMM models is based on the global estimation and they try to convert the segment amplitudes into very limited discrete transition states. As a result, when the data has varying segment amplitudes and some of them have short segment lengths, HMM models tend to have worse performance than other algorithms, like CBS and GLAD. On the other hand, the CBS and GLAD algorithms have consistent performance for varying and fixed segment amplitudes, as shown in Table **2**. Comparing HomHMM nad BioHMM, BioHMM has better overall performance, but it is at the expense of much longer running time. CBS comparatively has better performance than GLAD, but it is also slightly slower than GLAD.

## URGENT NEEDS FOR BENCHMARK DATA SETS

In the previous section, we have shown how to use different algorithms to analyze aCGH data. But which algorithm shall one choose? Different algorithms are usually based on different statistical models with different assumptions, but which one is closer to the reality? These questions cannot be easily answered with simulated data only.

Currently, biological plausibility has been always used as a validation of the findings. For instance, the amplified or deleted regions were always cross-referenced with previous cytogenetic studies. However, boundaries from cytogenetic data are of much lower resolution than aCGH data; it cannot be used to judge different algorithms that call the boundary in the difference of 10 Kb. Moreover, biological plausibility is only a weak and indirect evidence to show the effectiveness of an algorithm.

**Fig. (2):**
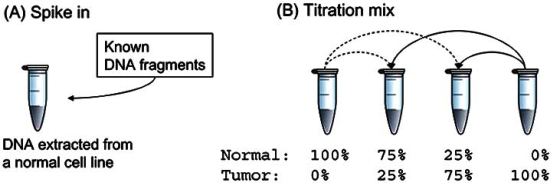
Data sets from necessary benchmark experiments to evaluate aCGH algorithms. (**A**) Spike-in and (**B**) Titration.

To move the data analysis forward and critically assess each method’s strength and weakness, we suggest that two types of benchmark data sets are urgently needed (Fig. **2**).

### 1) Spike-in Benchmarks

Similar to the Affymetrix Latin Square Spike-in data of mRNAs, several pieces of chromosomes can be spiked into a sample in permutations. Because of the truth is known, the algorithms can be explicitly tested for their performance on false positive and false negative rates.

### 2) Titration Benchmarks

A titration benchmark data is especially important for the study of tumor genetic alterations where the sample is more likely to be a mixture of normal and abnormal cell types. First, tumors are known to be genetically heterogeneous, where certain clones may harbor amplification and deletions at certain regions and others do not. Second, the samples measured usually contain adjacent normal cells. Although laser microdissection or fluorescence *in situ* hybridization offers a workaround, it is critical to understand how an analytical system is capable to handle admixtures. A titration experiment in Fig. (**2**) (B) using normal and tumor cell lines can facilitate the evaluation.

## RT-PCR VALIDATION OF THE HIGH-THROUGHPUT RESULTS

Similar to microarray-based RNA profiling, the results from aCGH study usually need to be cross-validated using another platform, for instance, conventional cytogenetics, FISH, or RT-PCR.

For RT-PCR based validation, a reference gene should be chosen to normalize the results. Generally, the reference gene should not have copy number variation in normal populations. Conceptually, it is very similar to “housekeeping” gene used for the RT-CPR study of mRNAs. For instance, the gene of RNA-specific adenosine deaminase (ADAR) was used as a reference gene in a glioma study [2] to validate the chromosomal loss or gain.

## Figures and Tables

**Fig. (1):**
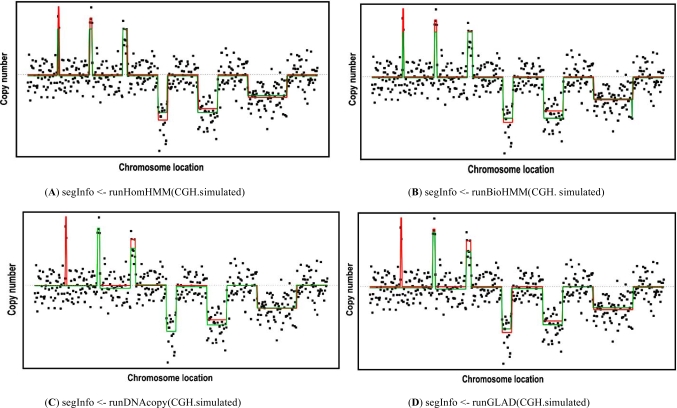
Examples of the aCGH segmentation using simulated aCGH data. The black dots are real simulated aCGH data, the red line is the true segmentation and the green line is the estimated segmentation result. Four different algorithms are used; the corresponding codes are shown below each figure, where “<-“ indicates an assignment to a variable and “CGH.simulated” is the simulated aCGH data.

**Table 1.** Free, Open-Source Bioconductor Packages for aCGH Analysis

Package	Algorithm	Major Features
aCGH	HomHMM	Homogeneous Hidden Markov Model
snapCGH	BioHMM	Heterogeneous Hidden Markov Model (transition probability depends on the distance between adjacent clones)
DNAcopy	CBS	Using circular binary segmentation
GLAD	GLAD	Break points detection based on the Adaptive Weights Smoothing

**Table 2.** Evaluation of aCGH Segmentation Algorithm Using Simulated Data

	Mean_Diff1_	SD_Diff1_	Mean_Diff2_	SD_Diff2_	Time (Seconds)
HomHMM	13.12	4.19	23.78	7.50	11.2
BioHMM	**6.84**	**3.77**	20.61	7.02	896.1
CBS	17.75	4.04	**17.38**	**4.39**	66.7
GLAD	19.94	4.25	19.63	4.39	29.0

“Diff1” represents the difference between the estimated and true segmentation for the fixed segmentation amplitudes, and  “Diff2” represents the one for varying segmentation amplitudes, as shown in Fig. (**1**). “Mean” represents the average of the difference, and  “SD” represents the standard deviation.  “Time” is the total time of processing 100 simulated aCGH data.
